# Occupational Health of Health-Care Workers with Overnutrition: Scoping Review with Meta-Analysis

**DOI:** 10.3390/nu15153416

**Published:** 2023-07-31

**Authors:** Cynthia Sarah Garibay-Lagos, Maria Isabel Martos-Boira, Elizabeth Landeta-Iza, Giselle Beatriz Contreras-González, Carmina Wanden-Berghe, Javier Sanz-Valero

**Affiliations:** 1 Occupational Risk Prevention Unit, Álvaro Cunqueiro Hospital, 36312 Vigo, Spain; cynthia.garibay@gmail.com; 2 Occupational Risk Prevention Unit, Hospital Universitario de Móstoles, 28935 Móstoles, Spain; imartosb@gmail.com; 3 Occupational Risk Prevention Service, Hospital San Pedro, 26006 Logroño, Spain; elizabethlandeta@yahoo.com; 4 Occupational Medicine Teaching Unit, Marqués de Valdecilla University Hospital, 39008 Santander, Spain; gisellebcg@gmail.com; 5 Foundation for the Promotion of Health and Biomedical Research in the Valencian Region (FISABIO), Health and Biomedical Research Institute of Alicante (ISABIAL), 03010 Alicante, Spain; carminaw@telefonica.net; 6 Carlos III Health Institute, National School of Occupational Medicine, 28029 Madrid, Spain

**Keywords:** overnutrition, obesity, overweight, occupational health, health personnel, review literature as topic

## Abstract

(1) Objective: To review the scientific literature on the impact of interventions to enhance the occupational health of health-care workers with overnutrition. (2) Methods: Scoping review with meta-analysis. Data were obtained by consulting the following bibliographic databases: MEDLINE (via PubMed), Embase, Cochrane Library, Scopus, Web of Science, Latin American and Caribbean Literature on Health Sciences (LILACS), and Medicina en Español (MEDES). The terms used as descriptors and as text in the title and abstract fields of the records were “health workers”, “overnutrition”, and “occupational health”, using the filters “human”, “adult”: 19+ years”, and “clinical trial”. The search update date was January 2023. The documentary quality of the articles was evaluated using the CONSORT questionnaire and the presence of bias was assessed using the Rob 2.0 tool. (3) Results: From the 611 digitally retrieved references, 17 clinical trials were selected after applying the inclusion and exclusion criteria. CONSORT scores ranged from a minimum of 14.6% to a maximum of 91.7%, with a median of 68.8%. According to the SIGN criteria, this review provided “1” evidence with a grade B recommendation. Six different types of intervention were tested, grouped into strategies ranging from a single intervention to a combination of four interventions. The summary effect of the meta-analysis showed significant weight loss, but no association with reduced body mass index. (4) Conclusions: While workplace interventions have been shown to be potentially effective, and strategies using different types of interventions have been proven to be useful in tackling overnutrition, an effective and sustainable solution for changing the behavior of health professionals to tackle overweight and obesity has yet to be identified.

## 1. Introduction

Overweight and obesity (overnutrition) have contributed substantially to a variety of chronic diseases including diabetes, cardiovascular diseases, hyperlipidemia, and arthritis [1]. Thus, it is recognized that morbidity related to overnutrition gives rise to indirect labor costs in terms of productivity loss due to both presenteeism [2] and absenteeism [3]. Understanding the factors tied to obesity in the workforce is therefore essential to developing effective interventions [4].

In addition, according to the work of Luckhaupt et al. [5] performed in the USA, health-care workers had a higher incidence of obesity. Estabrook et al. [6] reported that as a result of the high workload of health-care workers, they engage in stress-induced eating, especially during shift work. These poor nutritional habits lead these workers to both malnutrition and overnutrition [7].

If workplace health interventions are crucial to improving the health and well-being of workers and promoting healthy lifestyles [8], it may be hard to find ways to address obesity due to overnutrition in health-care workers. As Kelly & Wills [9] pointed out, there is insufficient evidence of appropriate interventions to address the problems of overnutrition in health-care workers.

In this regard, the systematic review conducted by Power et al. [10] concluded that interventions combining diet and physical activity had better outcomes, and Upadhyaya et al. [11] noted that interventions with multiple health-care components could be successful in enhancing workers’ weight and encouraged health professionals to continue working on the development of new interventions. By the same token, Melián-Fleitas et al. [12] stated that interventions involving more than one strategy have generally shown to be useful in overcoming overweight and obesity in the workforce.

There are research studies that have assessed dietary interventions in various groups of employees, reporting a beneficial effect on dietary outcomes [12,13]. Nevertheless, these studies are generally very heterogeneous in terms of study design, sample size, and intervention type. In the review carried out by Panchbhaya et al. [14], it was evidenced that some of the reviewed studies had a high level of heterogeneity and reported insufficient information to ascertain the possibility of bias.

Thus, the aim of this review was to re-appraise the scientific literature on the influence of interventions to improve the occupational health of health-care workers with overnutrition.

## 2. Materials and Methods

### 2.1. Design

A cross-sectional descriptive study and critical analysis of the retrieved papers using the systematic technique according to the extension for scoping reviews proposed through the PRISMA (Preferred Reporting Items for Systematic Reviews and Meta-Analyses) statement [15].

### 2.2. Source of Data Collection

Data were obtained from direct and access consultation, through the Internet, and from the following bibliographic databases in the health sciences field: MEDLINE (via PubMed), Embase, Cochrane Library, Scopus, Web of Science, Latin American and Caribbean Literature in Health Sciences (LILACS), and Medicina en Español (MEDES).

### 2.3. Information Processing

To delineate the search terms, the Thesaurus of Health Sciences Descriptors (DeCs) developed by the Latin American and Caribbean Centre on Health Sciences Information (BIREME) and its equivalence with the one established by the U.S. National Library of Medicine, the Medical Subject Headings (MESH), were consulted.

From the hierarchical study of both thesauruses and their indexing cards (Entry Terms), the following search equations were considered appropriate:

- Population: Health Personnel—Men and women working in the provision of health services, whether as individual practitioners or employees of health institutions and programs, whether professionally trained or not, and whether subject to public regulation or not.
  “Health Personnel”[Mesh] OR “Health Personnel”[Title/Abstract] OR “Health Care Provider*”[Title/Abstract] OR “Healthcare Provider*”[Title/Abstract] OR “Healthcare Worker*”[Title/Abstract] OR “Health Care Professional*”[Title/Abstract] OR “Nurse*”[Title/Abstract] OR “Pharmacist*”[Title/Abstract] OR “Physician*”[Title/Abstract] OR “Health Care Personnel”[Title/Abstract] OR “Health Care Practitioner*”[Title/Abstract] OR “Health Care Worker*”[Title/Abstract] OR “Health Profession Personnel”[Title/Abstract] OR “Healthcare Personnel”[Title/Abstract] OR “Healthcare Practitioner*”[Title/Abstract] OR “Healthcare Professional*”[Title/Abstract]
- Intervention: Overnutrition—An imbalanced nutritional status resulting from excessive intake of nutrients. Generally, overnutrition generates an energy imbalance between food consumption and energy expenditure, leading to disorders such as obesity.
  “Overnutrition”[Mesh] OR “Overnutrition”[Title/Abstract] OR “Hypernutrition”[Title/Abstract] OR “Overweight”[Title/Abstract] OR “Obesity”[Title/Abstract] OR “Hyperphagia”[Mesh] OR “Hyperphagia”[Title/Abstract] OR “Overeating”[Title/Abstract] OR “Polyphagia*”[Title/Abstract] OR “Dietary Excess”[Title/Abstract] OR “Excessive Feeding*”[Title/Abstract] OR “Hyper-Nutrition”[Title/Abstract] OR “Over-Nutrition”[Title/Abstract] OR “Overeating”[Title/Abstract] OR “Overfeeding”[Title/Abstract]
- Result: Occupational Health—The promotion and maintenance of physical and mental health in the work environment.
  “Occupational Health”[Mesh] OR “Occupational Health”[Title/Abstract] OR “Industrial Hygiene”[Title/Abstract] OR “Industrial Health”[Title/Abstract] OR “Occupational Safety”[Title/Abstract] OR “Employee Health”[Title/Abstract] OR “Occupational Exposure”[Mesh] OR “Occupational Exposure”[Title/Abstract] OR “Occupational Stress”[Mesh] OR “Occupational Stress”[Title/Abstract] OR “Occupational Disease”[Mesh] OR “Occupational Disease*”[Title/Abstract] OR “Occupational Hazard*”[Title/Abstract] OR “Occupational Medicine”[Mesh] OR “Occupational Medicine”[Title/Abstract] OR “Occupational Health Safety”[Title/Abstract] OR “Occupational Health Service*”[Title/Abstract] OR “Occupational Stressors”[Title/Abstract] OR “Occupational Factors”[Title/Abstract] OR “Workplace”[Mesh] OR “Workplace*”[Title/Abstract] OR “Workplace Health”[Title/Abstract] OR “Workplace Safety”[Title/Abstract] OR “Safety Climate”[Title/Abstract] OR “Total Worker Health”[Title/Abstract] OR “Working Environment”[Title/Abstract] OR “Job Satisfaction”[Mesh] OR “Job Satisfaction*”[Title/Abstract] OR “Job Stress”[Title/Abstract] OR “Job Security”[Title/Abstract] OR “Psychosocial Working Condition*”[Title/Abstract] OR “Employee Health”[Title/Abstract]

The final search equation was developed for use in the MEDLINE database, via PubMed, by the Boolean union of the 3 proposed equations: Population AND Intervention AND Outcome, using the filters: humans “Humans” and adults “Adult: 19+ years”.

This strategy was subsequently adapted to the characteristics of each of the other consulted databases, performing the search from the first available date in each of the selected databases until January 2023.

In addition, a supplementary search was performed to lessen the possibility of publication bias by manually searching the reference lists of the articles that were selected for the review and related systematic reviews. Moreover, the list of similar articles provided by MEDLINE was revised in each of the selected trials.

### 2.4. Final Selection of Articles

Articles that met the following criteria were selected for review and critical analysis:

- Inclusion: being a clinical trial, being published in peer-reviewed journals, and written in English, Spanish, and Portuguese.
- Exclusion: those articles for which the full text could not be found, there was no association between the intervention and the outcome under the study criterion of causality, and those that included a non-adult population (under 18 years of age).

The selection of the relevant articles was carried out by the authors of this review. To validate the inclusion of the articles, it was set that the concordance assessment of the selection should be greater than 0.60 [16]. Provided that this condition is fulfilled, possible discrepancies were resolved by consensus among all the authors of this review.

### 2.5. Documentary Quality, Level of Evidence and Recommendation, and Study of Biases

The appropriateness of the selected articles was assessed using the CONSORT (CONsolidated Standards Of Reporting Trials) guidelines for reporting observational studies [17], which contains a list of 25 essential checkpoints to be described during the publication of these papers. For each selected article, one point was assigned for each present item (if not applicable, no score was given). When an item was made up of several sections, these were assessed independently, giving the same value to each of them, before being averaged (this being the final result for that item). Thereby, in no case was the total score of one point exceeded.

The Scottish Intercollegiate Guidelines Network Grading Review Group (SIGN) [18] was used to determine the level of evidence and recommendation.

The tool modified by RoB.2 [19,20] was utilized to assess the potential biases of the trials included in the review: bias was evaluated using the criteria of high, low, or doubtful bias for the dimensions: D1 Bias arising from the randomization process, D2 Bias due to deviations from intended intervention, D3 Bias due to missing outcome data, D4 Bias in measurement of the outcome, and D5 Bias in selection of the reported result.

The publication bias study was carried out using the Funnel Plot graphics [21].

### 2.6. Data Extraction

The control of data extraction was performed using double tables that allowed the detection of digressions and their rectification by re-consulting the originals.

The refinement of duplicate records (records present in more than one database) was conducted using the multiplatform program ZOTERO (a bibliographic reference manager developed by the Center for History and New Media at George Mason University).

The Burton–Kebler half-period (BK) and the Price index (PI) were calculated to determine the timeliness of the studies.

The articles were gathered according to the variables under study to systematize and streamline the understanding of the outcomes. To do so, the following data were considered: first author, year of publication, studied population, declared pathology, country and period of the study, performed data, and main result motivated by the effect of the action.

### 2.7. Data Analysis

Data related to information retrieval were presented in terms of frequency and percentage.

To determine the BK, the median age was calculated regarding the time range analyzed, and the PI was computed by the percentage of articles with an age of less than 5 years. The measure concordance was performed using IK to ascertain the adequacy of the selection of articles. The relationship between authors was considered well-founded when its value was greater than 60% (good or very good concordance strength).

The CONSORT questionnaire scores were analyzed using the median, maximum, and minimum scores. The evolution of these grades over the years of publication was procured using Pearson’s correlation analysis.

For the meta-analysis, we employed the standardized mean effects technique with Hedges’ g and the Knapp–Hartung adjustment. In addition, the inter-study variability estimated with the between-study variance τ2 and its statistical significance using Wald’s Q was used to assess heterogeneity.

Data analysis was performed through R v4.22 software with the RStudio 2022.10.0 build 353 work package, the specific library used to calculate the risk of bias was “robvis” v0.3.0.900 while the specific library utilized for meta-analysis was “meta” v6.1-0.

### 2.8. Ethical Aspects

All data were obtained from the accepted articles for review. Thus, in accordance with Law 14/2007 on biomedical research [22], the approval of the ethics committee was not required when using secondary data.

## 3. Results

Having applied the search criteria, a total of 611 references were retrieved: 27 (4.42%) from MEDLINE (via PubMed), 11 (1.80%) from Embase, nine (1.47%) from Cochrane Library, 532 (87.07%) from Scopus, and 32 (5.24%) from Web of Science. No papers were retrieved from the LILACS and MEDES bibliographic databases. Consultation of the bibliographic lists of the selected articles resulted in the selection of 13 studies.

**Figure 1:**
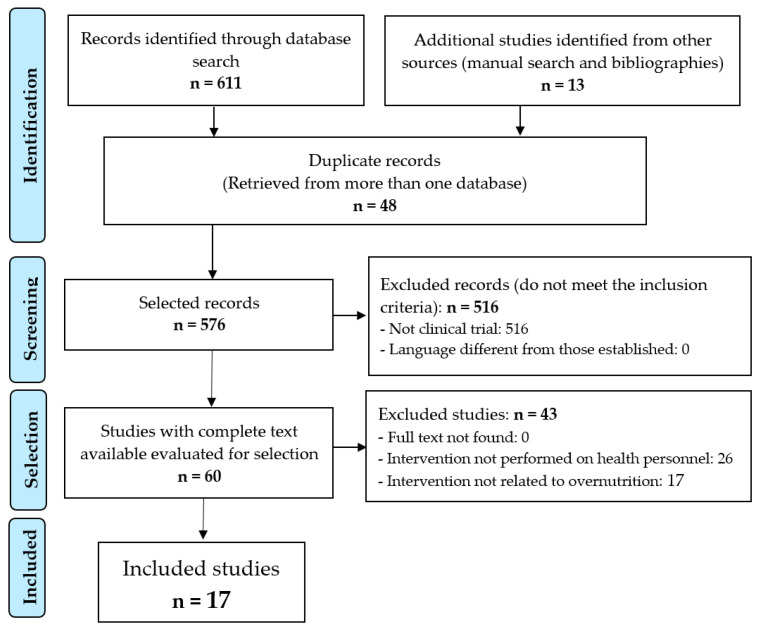
Diagram of the selection procedure of the studies.

After filtering out the repeated records and applying the inclusion and exclusion criteria (Figure 1), it was possible to select 17 papers [23,24,25,26,27,28,29,30,31,32,33,34,35,36,37,38,39] for review and critical analysis; see Table 1.

Agreement on the pertinence of the selected studies among the reviewers, calculated using the Kappa index, was 68.30% (*p* < 0.01).

The clinical trials selected for the review were randomized in 16 cases, of which 15 were randomized in parallel [21,22,23,24,26,27,28,30,31,32,33,34,35,36,37], while the paper carried out by Leedo et al. [25] was a randomized crossover trial. The study conducted by Speroni et al. [29] was not randomized.

According to the Burton–Kebler Index equal to 10.0 years, the selected articles demonstrated an obsolescence with a Price Index of 7.4%. The years with the highest number of published papers were 2017 and 2012, in which three articles were chosen for the review [23,24,25,28,29,30].

When assessing the adequacy of the studies using the CONSORT guidelines, the percentages of compliance ranged from a minimum of 14.6% to a maximum of 91.7%, with a median of 68.8%. A good direct exponential trend was observed (R2 = 0.63; *p* < 0.001). Item 18 was not used since it was not included in the secondary analysis. In the study conducted by Speroni et al. [29], items 8, 9, 10, and 11 were not applied as there was no randomization, see Table 2.

Based on the SIGN criteria, this review presented evidence with a grade of 1 (systematic review of randomized clinical trials or randomized clinical trials at high risk of bias) and with a grade of recommendation B (a body of evidence that encompasses studies that are directly applicable to the target population and showed overall consistency of outcomes or extrapolation of studies).

**Figure 2:**
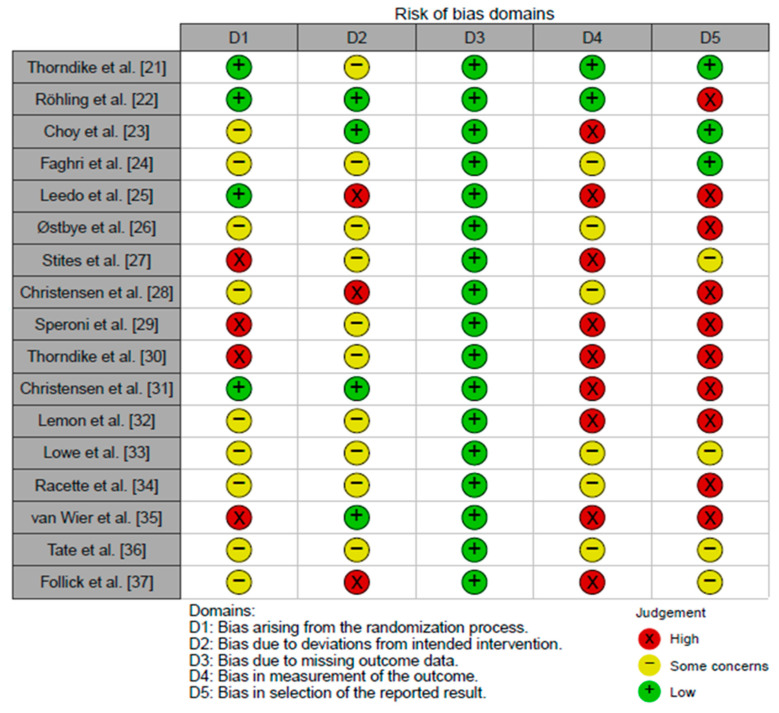
Assessing the methodological risk of clinical trials reviewed using the RoB.2 tool [21,22,23,24,25,26,27,28,29,30,31,32,33,34,35,36,37].

The RoB.2 tool, which assesses the methodological risk of bias, was used to assess the examination of bias in the trials included in the review, as shown in Figure 2.

**Figure 3:**
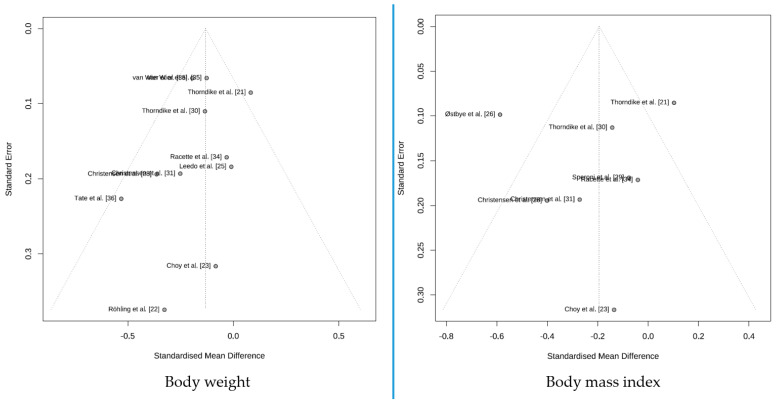
Funnel plot for publication bias study [21,22,23,24,25,26,27,28,29,30,31,32,33,34,35,36,37].

The funnel plot shows that publication bias was not particularly pronounced for body weight or body mass index, see Figure 3.

The study with the largest population was performed by Van Wier et al. [35], with N = 1386 health-care workers, while the study with the smallest population was conducted by Stites et al. [27], with 26 employees. This population was mostly female, except in the study carried out by Van Wier et al. [35], albeit four (23.53%) studies did not report the male/female ratio [23,24,34,37]. The participants were overweight or obese in 14 out of 17 trials, although one trial selected an obese population [26] and two studies worked with an overweight population [35,37].

Stites et al. [27] conducted the study with the highest mean age in the intervention group, with a mean age of 48.6 ± 11.6, whereas Tate et al. [36] carried out the trial with the lowest mean age in this group, with 41.1 ± 11.6. Regarding the control group, the paper with the highest mean age was that of Röhling et al. [22] with 49 ± 7 years and the study with the lowest mean age was that of Tate et al. [36] with 40.6 ± 9.7 years.

There were five clinical trials [23,24,33,34,37] that did not report the mean ages of either the intervention or control groups. Furthermore, Østbye et al. [26] reported an age range while Lemon et al. [32] listed the percentage of employees who were in a specific age range.

The United States was the largest contributor with 11 (64.7%) papers [21,24,26,27,29,30,32,33,34,36,37], followed by China with one (5.9%) [23]. There were five studies with a European affiliation (29.4%) [22,25,28,31,35].

The intervention period ranged from a minimum of 8 weeks [23,25] to a maximum of 2 years [21,32], with one year being the most common intervention period [26,28,31,33,34].

### 3.1. Types of Interventions Observed

The interventions gathered from the retrieved clinical trials were:

A. Nutrition education and healthy lifestyles:
  - Pre-order, with/without nutrition information
  - Nutrition education sessions
  - Exercise and nutrition resources
  - Nutrition education sessions, color-coded food labeling
  - Healthy snacks, nutrition seminars
  - Information on nutrition and physical activity, strategies for lifestyle modification
  - Weight loss session, online resources.

B. Behavioral Intervention
  - Comments on previous purchases in the coffee shop
  - Peer comparisons
  - Cognitive behavioral training
  - Social marketing campaign
  - Strategies to foster interpersonal support
  - Behavioral lessons: structural guidance on a variety of weight loss topics: nutrition, exercise, or behavioral self-regulation strategies.

C. Diet
  - Low-carbohydrate nutrition and meal replacement therapy
  - Diet
  - Cold food, water bottle, snack
  - Dietary recommendations
  - Meal distribution
  - Individual dietary plan
  - Environmental strategies to promote a healthy diet.

D. Physical exercise
  - Telemetric devices to measure your weight and steps steadily
  - Physical activity
  - Pedometer, exercise session, yoga
  - Strengthening exercises, aerobic exercise
  - Environmental strategies to foster physical activity.

E. Economic intervention
  - Financial incentives for healthier purchases
  - Direct cash input for weight loss
  - Discounts on food at the workplace cafeteria
  - Bestowing rewards for getting involved in different activities—co-food and exercise equipment of varying values
  - Loss of money if the objectives have not been achieved, and a financial gain if the targets have been accomplished.

F. Coaching
  - Telemedical coaching
  - Coaching sessions
  - Mindful eating training
  - Standardized counseling

The strategies reported in the clinical trials reviewed ranged from 1 to 4 possible interventions, as shown in Table 3.

### 3.2. Results Procured from the Interventions Performed

The results of 10 of the 17 reviewed clinical trials [21,22,23,24,26,27,28,29,30,31,33,34,35,36,37] demonstrated that the interventions reduced the mean weight or BMI of the intervention group, albeit this decrease was not always statistically significant. Additionally, there were clinical trials (21,32,33) in which no weight or BMI loss was observed, but rather an increase in the final mean weight or BMI of the intervention group. It is noteworthy that in some trials it was not possible to obtain data on weight [24,26,27,29,32,37] or BMI [24,25,27,32,33,35,36,37].

Educational intervention and habit change evidenced significant results in six clinical trials [22,24,28,29,31,34]. Positive outcomes were also found for coaching interventions in six clinical trials [22,23,26,30,31,35]. Christensen et al. [28] reported satisfactory results with applied cognitive behavioral training, while Van Wier et al. [35] used various means of communication and support to achieve weight loss.

With great potential but still needing to be developed, five trials [21,24,27,33,37] of economic interventions were reviewed and significant outcomes were procured. Nevertheless, this type of intervention was successful in the short term but could not be sustained over the long term [24].

### 3.3. Results Obtained from the Developed Strategies

Clinical trials of single intervention found no meaningful change in weight with either dietary [25] or economic [37] interventions. Meaningful changes were only observed with interventions in nutrition education and healthy lifestyle [23], with a net difference in mean BMI of −0.4 in the intervention group.

Regarding the trials that combined two interventions, it is worth noting that all of them included nutrition education and a healthy lifestyle. However, the inclusion of physical activity [30] demonstrated no association with weight loss, although these trials showed moderate weight loss and dietary improvement. The inclusion of economic intervention [33] also indicated no association. Furthermore, the study that assessed the behavioral intervention (a structured behavioral therapy program with weekly contact and individualized feedback via the Internet) [36] found a significant association with weight loss in participants (*p* < 0.05).

In trials combining three interventions, several found no meaningful difference in weight loss [21,26,27,32], whilst others found a significant difference [24,28,29,31,34,35]. However, it is noteworthy that the study conducted by Lemon et al. [32] showed a dose-response relationship when intervention exposure was weighed as an independent variable: for each increase in intervention participation, there was a 0.012 unit decrease in BMI from baseline to 24 months (IC del 95% = −0.025–0.001).

In the trial that combined four interventions (nutrition education and healthy lifestyle, diet, exercise, and coaching) [22], there was weight reduction in the intervention group, as well as improvements in other parameters such as fasting blood glucose, HbA1c, quality of life, fasting insulin, blood pressure, and eating demeanor (all *p* < 0.05).

### 3.4. Results from the Meta-Analysis

**Figure 4:**
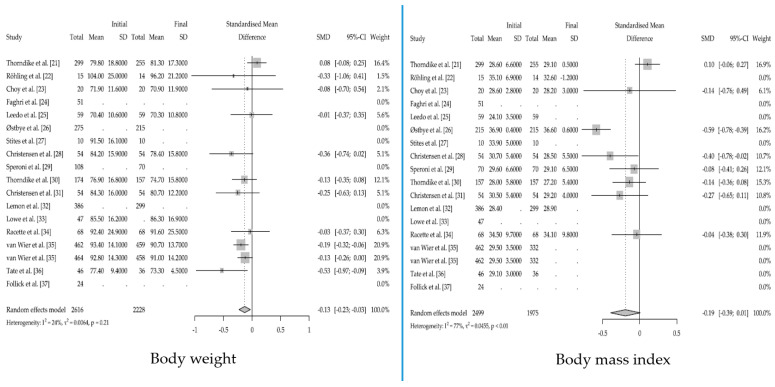
Forest plot of clinical trials reviewed for weight and body mass index [21,22,23,24,25,26,27,28,29,30,31,32,33,34,35,36,37].

The change in body weight and body mass index in the reviewed trials is shown in Table 4. The effect sizes calculated from the meta-analysis are shown in Figure 4.

When analyzing the results for body mass index, the heterogeneity was 77% (*p* < 0.01), decreasing to 24% (*p* = 0.21) when body weight was studied. When analyzing the results for body weight, the null hypothesis of homogeneity could be accepted.

It should be noted that when reviewing the results for body mass index, there were trials [24,25,27,32,33,36,37] with missing data, and when reviewing the results for body weight, there were six studies [24,26,27,29,32,37] with missing data.

## 4. Discussion

Given the recommendations on the objectives of a systematic review [38], the present review collects relevant information on the occupational health of health-care employees with overnutrition, with the aim of providing the scientific community with evidence that can help to foster effective interventions to protect workers’ health. Moreover, the review is in line with the World Health Organization’s strategy, which emphasizes the importance of setting primary prevention and interventions for the enhancement of occupational health [39].

The obsolescence of the reviewed articles was comparable to that found in previous reviews on nutrition and occupational health [8,12]. This is because the mean age of the reviewed studies exceeds what is expected in the health sciences and highlights the need for updating.

The assessment through the CONSORT criteria was superior to that observed in recent systematic reviews on occupational health [8,12,40]. The study of the time trend in the appropriateness of the papers included in the review was to be expected, since the first papers using these criteria date back to 1996 [41], and their use has been shown to be ongoing. In addition, Turner et al. [42] demonstrated that the adoption of these criteria has led to enhancements in the quality of articles.

The level of evidence and recommendation of this study, as determined by the SIGN criteria, was akin to previous studies, although some studies were more biased than others and therefore the conclusions were weaker [43]. The conclusions of many occupational health studies are still not based on the best available evidence [44]. This may be due to the experimental design of primary studies, such as clinical trials, which although robust, may not be appropriate for evaluating occupational health interventions because they tend to have very long-term effects; or, as in this review, the interventions were not specifically measured in the field of workers’ health. Regardless, this review provides a sufficient body of evidence and includes studies that are directly applicable to the target population and show overall consistency of results.

Using the Rob 2.0 tool, the evaluation of the potential bias of the trials included in the review confirmed what was discussed in the previous paragraph as well as what was observed in the assessment using the CONSORT form.

Given that the study population was predominantly female, this is in line with the World Health Organization document “Gender equity in the health workforce: analysis of 104 countries” [45], which states that 70% of health and social workers (the target population of this review) are women. The age of this population was within the expected range for health workers.

The predominance of American affiliations is well-known and widely reported in the scientific literature. The prestige of its universities and the substantial public and private funding of its institutions and research centers contribute to this [46]. However, there are more important reasons that justify research into overnutrition in this country. For instance, the prevalence of obesity in the adult population is 41.9%, with the incidence of severe obesity at 9.2%. This has driven increases in heart disease, stroke, and type 2 diabetes, which are the leading causes of premature and avoidable death [47].

The follow-up period in some trials [23,25] was considered too short (only 8 weeks) to assess the outcomes of the intervention, and a period of several weeks or even months is deemed necessary to assess the results and to see the follow-up of the intervention. Weight loss, as shown in this review, can be achieved by a variety of interventions, but long-term maintenance of weight loss is much more difficult. As noted by Hall and Kahan [48], obesity interventions generally result in rapid and early weight loss, followed by a weight plateau and progressive weight regain. Thus, the treatment of obesity and overweight requires ongoing clinical care and specific counseling to support sustainable healthy behaviors.

In the analyzed interventions, nutrition education and healthy lifestyle approaches were seen as the basis for weight reduction. This has already been described in the work of Thorndike et al. [49], who included food labeling (traffic light colors) and found an association with a sustained decrease in purchased calories, particularly from unhealthy foods, by hospital staff, which might help in improving dietary intake and preventing obesity among staff. Nonetheless, the work of Braeckman et al. [50] found changes in nutritional knowledge and a decrease in total calorie intake in the workplace, but no significant change in body weight.

Regarding behavioral interventions, the review conducted by Hartmann-Boyce et al. [51] also noted the substantial heterogeneity of behavioral interventions, both in terms of program content and treatment outcomes, highlighting six components that showed clinically significant benefits, one of which was dietary modification: this involved offering partial or complete meal replacements. This was also found in this review.

This is similar to the article by Hilbert et al. [52], which concluded that group cognitive remediation therapy did not improve weight loss in adults with obesity in comparison with no treatment prior to behavioral therapy for weight loss. However, a current meta-analysis of the effectiveness of acceptance and commitment therapy showed that it may be effective in improving weight loss in terms of BMI [53].

In terms of dietary intervention, it was found that, as in this review, previous studies showed a statistically meaningful decrease in weight [54], while others did not show this change in body weight [50].

As documented in the results of the present paper, studies using exercise strategies only have reported mixed results [55,56].

In the cost-effective interventions, Kullgren et al. [57] showed significant weight loss, which was maintained over time in the intervention group. On the other hand, Follick et al. [37] concluded that participants did not maintain their weight loss at the 6-month follow-up. Vermeer et al. [58] assessed eating behavior after exposure to the sale of smaller portions at discounted prices, and while some consumers were inclined to choose smaller portions, the discounted prices had no additional effect.

Although coaching applied to health interventions has emerged as a supportive tool to overcome behavioral barriers, the review of Sieczkowska et al. [59] concluded that the available evidence was not sufficient to endorse its use as a health-care intervention for weight loss. This was not the case for Sforzo et al. [60], who disagreed with this statement.

Regarding the different strategies observed, it was found that those that included three or four interventions had a greater decrease in body mass index, with the interventions included in these strategies being mainly: nutrition education and healthy lifestyles, physical activity, and diet. These findings were also observed by Anderson et al. [55], who reviewed the effectiveness of diet and exercise interventions in the workplace and reported a slight reduction in terms of weight change. Another review, not aimed specifically at health-care workers, reviewed 13 clinical trials and found that weight was significantly reduced in the intervention groups, indicating that educational and behavioral strategies combined with physical activity interventions led to weight loss [10]. Upadhyaya et al. [11] reviewed 51 workplace interventions (most of which were related to diet and exercise) and found that combining behavioral interventions resulted in better outcomes. This means that multi-component strategies may have a greater effect on weight loss.

By the same token, according to the article by Muto and Yamauchi et al. [61], a multi-component employee health promotion program has already been shown to be effective in improving obesity, hypertension, and hyperlipidaemia.

On the contrary, Vermeij et al. [56] quantified the impact of the social environment component in terms of its effectiveness in reducing body weight. For instance, Racette et al. [34] tested the adequacy of team skills and Muto et al. [61] demonstrated the importance of family involvement. Therefore, future studies should consider social components alongside other workplace interventions [62].

Another important issue, not observed in the reviewed works, was healthy lifestyles. Thus, the study by Rapisarda et al. [63] on the evaluation of a joint health promotion intervention in a cohort of health workers, who had at least one cardiovascular risk factor, highlighted the importance of using multidisciplinary approaches when planning workplace interventions, as already verified in this review. Therefore, intervention studies on hypernutrition should take into account healthy lifestyles.

### 4.1. Critical Analysis

Notwithstanding the existence of overnutrition (obesity and overweight) and the known prevalence of obesity and related diseases, no effective strategies have been identified to lessen it. For example, over the past 30 years, no World Health Organization member country has been able to reverse the trend of increasing obesity and/or overweight in the population, including the working population [64].

Hence, according to Kunyahamu et al. [65], more efforts are needed to understand the factors that may contribute to overnutrition among health-care workers and to implement “effective” strategies to tackle overnutrition, primarily targeting health-care workers at higher risks of obesity. New approaches, some based on Web 2.0, are seen as crucial to reversing this situation.

### 4.2. Limitations

The results were limited by the shortcomings of each of the trials included in the review. Most of the trials did not state whether they controlled for confounding factors that could affect the outcomes. This confirms the moderate prevalence and level of recommendation found.

Conversely, many articles were retrieved from the Scopus and Web of Science databases that were ultimately irrelevant, which could be due to the lack of indexing (the search was performed in text format by consulting the title, abstract, and keywords) and the impossibility of limiting the search by article type. This high document “noise” has been reported in previous systematic reviews [46,66,67].

Another limitation was the scarcity of articles found and their lack of timeliness. This small number of articles may indicate that the search equation was too specific, raising doubts about possible documentary silence. Nevertheless, the manual search of the bibliographic lists of the included articles did not provide any new input for inclusion in the review.

Despite the current review finding a high risk of bias, previous reviews have also evidenced this to be the case. Thus, Allan et al. [68] mentioned the occurrence of bias in dietary interventions in workers. This situation was also reported in the review by Panchbhaya et al. [14]. In any case, this circumstance does not hinder knowledge about the effectiveness of strategies to reduce overnutrition among health-care workers.

Indeed, the lack of data in some clinical trials in this review may have hindered the availability of higher-quality evidence.

## 5. Conclusions

While workplace interventions have been shown to be potentially effective, and strategies using different types of interventions have been proven to be useful in tackling overnutrition, an effective and sustainable solution for changing the behavior of health professionals to tackle overweight and obesity has yet to be identified.

## Figures and Tables

**Table 1:** Summary of accepted articles for the review on occupational health of health personnel with overnutrition.

Author/Year	Population Studied	Country	Pathology	Intervention Period	Type of Intervention	Observed Outcome
Thorndike et al. 2021 [23]	N = 602 **IG:** n = 299 M/W = 69/230 Age Mean = 43.5 ± 12 years BMI = 28.6 ± 6.6 **CG:** n = 303 M/W = 55/248 Age Mean = 43.8 ± 12.5 years BMI = 28.0 ± 6.5	USA	Overweight and obesity	2 years	**IG:** Participants received two emails per week with feedback on previous cafeteria purchases and personalized health and lifestyle tips and one letter per month with peer comparisons and financial incentives for healthier purchases. **CG:** Participants received one letter per month with general healthy lifestyle information.	There were no between-group differences in weight change. IG increased green-labeled purchases and decreased red-labeled and calories purchased compared with CG (*p* < 0.001). The findings suggest that an automated behavioral intervention using workplace cafeteria data improved employees’ food choices but did not prevent weight gain.
Röhling et al. 2020 [24]	N = 30 **IG:** n = 15 M/W = 3/12 Age Mean = 44 ± 9 years BMI = 35.1 ± 6.9 **CG:** n = 15 M/W = 2/13 Age Mean = 49 ± 7 years BMI = 32.8 ± 6.1	Germany	Overweight and obesity	12 weeks	**IG:** Received seminars, low-carbohydrate nutrition including formula diet, continuous glucose monitoring, telemetric monitoring, and telemedical coaching) with weekly contacts. **CG:** Continued their habitual lifestyle. All participants were equipped with telemetric devices (scales and pedometers).	IG significantly reduced weight (*p* < 0.001) and improved in BMI, WC, fat mass, and all variables of eating behavior (all *p* < 0.05) compared to the CG.
Choy et al. 2017 [25]	N = 42 **IG:** n = 20 M/W = Not provided Age Mean = Not provided BMI = 28.59 ± 2.78 **CG:** n = 22 M/W = Not provided Age Mean = Not provided BMI = 28.9 ± 3.67	China	Overweight and obesity	8 weeks	**IG:** Received tailored weight management intervention, including individual nutrition counseling, nutrition pamphlets, telephone counseling, and smartphone text messages for eight weeks. **CG:** Received individual nutrition counseling and nutrition pamphlets. Both groups received a face-to-face education session for 45 min.	In comparison with the control group, the mean net weight loss in the intervention group increased at the end of the study. In the follow-up visit at week 8, the mean change in weight from baseline was −0.3 kg (95% CI) in the CG and −0.98 kg (95% CI) in the IG. The net difference in mean BMI in the intervention groups was −0.4 (95% CI).
Faghri et al. 2017 [26]	N = 99 IG: n = 51 M/W = Not provided Age Mean = Not provided BMI = Not provided **CG:** n = 48 M/W = Not provided Age Mean = Not provided BMI = Not provided	USA	Overweight and obesity	16 weeks	**IG:** Financial incentive-based intervention. All participants received a personalized weight loss consultation based on their reported physical activity habits and dietary preferences. Each participant received an action plan based on the National Diabetes Prevention Program (NDPP). **CG:** No incentive.	IG reduced more weight (*p* = 0.027) and BMI (*p* = 0.043) than CG at week 16. At week 28, IG lost more weight than CG (*p* = 0.053) and reduced their BMI more than CG (*p* = 0.308). Eating and exercise self-efficacy were significant mediators between health behaviors and weight loss (*p* < 0.05). Incentives significantly moderated the effects of self-efficacy (*p* = 0.00) on weight loss. Self-efficacy and financial incentives may affect weight loss and play a role in weight-loss interventions.
Leedo et al. 2017 [27]	N = 59 **IG:** n = 59 M/W = 7/52 Age Mean = 45.1 ± 9.3 years BMI = 24.1 ± 3.5 **CG:** n = 59 M/W = 7/52 Age Mean = 45.1 ± 9.3 years BMI ± SD = 24.1 ± 3.5	Denmark	Overweight and obesity	8 weeks	**Intervention period** (4 weeks): Received a keyhole-labeled meal, snack, and bottled water during each shift. **Control period** (4 weeks): Instructed to continue with their habitual dietary intake.	The intake of fat (*p* = 0.03) and polyunsaturated fatty acid (*p* = 0.003) was lower, and the intake of carbohydrate (*p* = 0.008), dietary fibre (*p* = 0.031), and water (*p* < 0.001) was greater in the intervention period than in the control period.
Østbye et al. 2015 [28]	N = 550 **IG:** (WM+ behavioral): n = 275 M/W = 45/230 Age: ≤50 years = 175 >50 years = 100 BMI = 37.37 ± 6.61 **CG:** (WM educational): n = 275 M/W = 48/227 Age: ≤50 years = 187 >50 years = 88 BMI = 37.02 ± 6.14	USA	Obesity	1 year	**WM+**: Intensive behavioral intervention: (1) monthly counseling sessions, (2) meetings with an exercise physiologist, (3) quarterly biometric feedback, (4) targeted health education materials, (5) information and active linking with various Duke programs and wellness resources, and (6) use of eHealth trackers for diet and weight. **WM:** Educational program targeting healthy lifestyle changes for weight loss (portion control, education, healthy diets, and physical activity).	There were no statistically meaningful differences between groups but there were modest reductions in BMI.
Stites et al. 2015 [29]	N = 26 **IG:** n = 10 M/W = 1/9 Age Mean = 48.6 ± 11.6 years BMI = 33.9 ± 5.0 **CG:** n = 16 M/W = 2/14 Age Mean = 42.6 ± 9.6 years BMI = 33.1 ± 11.2	USA	Overweight and obesity	12 weeks	**IG:** Baseline (4 weeks): participants selected their lunches as usual from the cafeteria. Full intervention (4 weeks): mindful eating training, pre-ordered lunches, price discounts. Partial intervention (4 weeks): pre-ordered lunches without price discounts. **CG:** Baseline (8 weeks), full intervention (4 weeks), and partial intervention (4 weeks).	The IG purchased lunches with an average of 144.6 fewer kilocalories (*p* = 0.01) and 8.9 fewer grams of fat (*p* = 0.005) compared to controls. Participants decreased their body weight from the beginning to the end of the study by an average of 0.40 kg.
Christensen et al. 2012 [30]	N = 98 **IG:** n = 54 M/W: 0/54 Age Mean = 45.7 ± 8.7 years BMI = 30.7 ± 5.4 **CG:** n = 44 M/W: 0/44 Age Mean = 46.0 ± 8.6 years BMI = 30.4 ± 4.9	Denmark	Overweight and obesity	1 year	**IG:** One-hour weekly workplace intervention consisting of diet, physical exercise, and cognitive behavioral training. **CG:** Monthly two-hour oral presentation during working hours about the Danish Dietary recommendations and other health-related topics.	The intervention generated substantial reductions in body weight (*p* < 0.001), BMI (*p* < 0.001), and body fat percentage (*p* < 0.001). The positive results support the workplace as an efficient arena for weight loss among overweight females.
Speroni et al. 2012 [31]	N = 217 **IG:** n = 108 M/W = 2/106 Age Mean (range) = 47.6 (22–67) years BMI = 30.5 ± 6.8 **CG:** n = 109 M/W = 7/102 Age Mean (range) = 45.2 (22–67) years BMI = 27.6 ± 5.3	USA	Overweight and obesity	24 weeks	**IG:** Included exercise (12 weekly sessions), yoga and nutrition (4 monthly sessions), and diary completion (exercise/yoga, food/water consumption, and sleep), addressing healthy lifestyle principles. **CG:** There were no other procedures for the contrast group. All participants completed an evaluation form about exercises and overall health.	IG experienced a greater mean reduction from baseline to week 12 in BMI than CG (IG = −0.494, CG = −0.180). This reduction in BMI among IG was significant based on a 1-tailed *t* test (*p* < 0.05). IG experienced a greater mean reduction in waist circumference (IG = −0.895, CG = -0.091) (*p* < 0.001) from baseline to week 12.
Thorndike et al. 2012 [32]	N = 330 **IG:** n = 174 M/W: 17/157 Age Mean = 44.2 ± 11.8 years BMI = 28.0 ± 5.8 **CG:** n = 156 M/W: 28/128 Age Mean = 41.6 ± 13.6 years BMI = 27.5 ± 5.9	USA	Overweight and obesity	10 weeks	Ten-week exercise and nutrition program (IG and CG) immediately followed by a 9-month maintenance intervention. **IG:** Internet support with a website for goal-setting and self-monitoring of weight and exercise plus minimal personal support (for 9 months). **CG:** Usual care (for 9 months).	The initial program resulted in moderate weight loss and improvements in diet and exercise behaviors at 1 year (*p* < 0.001) in both groups, but no difference in weight loss between groups. The Internet-based maintenance program immediately after did not improve these outcomes.
Christensen et al. 2011 [33]	N = 144 IG: n = 76 M/W: 1/75 Age Mean = 44.8 ± 9.5 years BMI = 28.4 ± 6.0 **CG:** n = 68 M/W: 4/64 Age Mean = 46.4 ± 9.5 years BMI = 27.8 ± 5.6	Denmark	Overweight and obesity	1 year	**IG:** An individual dietary plan with an energy deficit of 1200 kcal/day, strengthening exercises, and cognitive behavioral training during working hours for 1 h/week. Leisure time aerobic fitness was planned for 2 h/week. **CG:** Monthly oral presentations.	The significantly reduced body weight, body fat, waist circumference, and blood pressure as well as increased aerobic fitness in the intervention group (*p* ≤ 0.001) show the great potential of workplace health promotion among this high-risk workgroup.
Lemon et al. 2010 [34]	N = 806 **IG:** n = 386 M/W = 21.7%/78.3% Age: ≤50 years = 67.0 %>50 years = 33.0% BMI: <25 = 36.9% ≥25.0 = 63.1% **CG:** n = 303 M/W = 15.8%/84.2% Age: ≤50 years = 70.5% >50 years = 29.5% BMI: <25 = 31.6% ≥25.0 = 68.4%	USA	Overweight and obesity	2 years	**IG:** The intervention was designed to promote organizational and social norms related to healthy eating and physical activity in the worksite. **CG:** The control condition received no intervention.	Employees in intervention sites reported significantly greater improvements in perceptions of organizational commitment to employee health at 12 and 24 months compared to control sites, but there was no impact of the intervention on change in BMI from baseline to 12 (beta = 0.272; 95% CI = −0.271, 0.782) or 24 months (beta = 0.276; 95% CI = −0.338, 0.890) in intention-to-treat analysis.
Lowe et al. 2010 [35]	N = 96 **IG:** n = 47 M/W: 7/40 Age Mean = Not provided BMI = Not provided **CG:** n = 49 M/W: 11/38 Age Mean = Not provided BMI = Not provided	USA	Overweight and obesity	1 year	**IG:** Environmental change plus pricing incentives for purchasing low-energy-density foods. Education sessions about low-energy-density eating. **CG:** Environmental change (introduction of ten new low-energy-density foods and food labels).	There was no statistically significant change in weight during the cafeteria monitoring phase in either intervention condition, when controlling for baseline weight (*p* = 0.11).
Racette et al. 2009 [36]	N = 123 **IG:** n = 68 M/W: Not provided Age Mean = Not provided BMI = 34.5 ± 9.7 **CG:** n = 55 M/W: Not provided Age Mean = Not provided BMI = 31.1 ± 7.2	USA	Overweight and obesity	1 year	**IG:** Assessment and intervention (promotion of physical activity and favourable dietary patterns using pedometers, healthy snack cart, weight watchers’ meetings, exercise classes, seminars, and team competitions and rewards). **CG:** Assessment only. All participants received personal health reports.	Improvements (*p* ≤ 0.05) were observed in both groups for fitness, blood pressure, and total, HDL, and LDL cholesterol. Additional improvements occurred in the IG in BMI, fat mass, Framingham risk score, and prevalence of metabolic syndrome; only the changes in BMI and fat mass were different between groups.
van Wier et al. 2009 [37]	N = 1386 **IG phone:** n = 462 M/F = 321/141 Age Mean = 43 ± 8.8 years BMI = 29.5 ± 3.5 **IG internet:** n = 464 M/W = 302/162 Age Mean = 43 ± 8.4 years BMI = 29.6 ± 3.4 **CG:** n = 460 M/W = 306/154 Age Mean = 43 ± 8.7 years BMI = 29.6 ± 3.7	Overweight	Netherlands	6 months	**IG phone:** Received self-help materials, a lifestyle intervention program (10 modules about nutrition and physical activity), and phone counseling. **IG internet:** Received self-help materials and e-mail counseling. **CG:** Received only the self-help materials and no counseling.	The phone IG had a significant weight loss of 1.5 kg (95% CI −2.2; −0.8) in comparison with the CG. For the internet IG, this was 0.6 kg (95% CI −1.3; −0.01). The difference between the intervention groups was not statistically significant as their coefficients were mutually included in their 95% confidence intervals.
Tate et al. 2001 [38]	N = 91 **IG:** n = 46 M/W = 5/41 Age Mean = 41.1 ± 11.6 years BMI = 29.1 ± 3.0 **CG:** n = 45 M/W = 5/40 Age Mean = 40.6 ± 9.7 years BMI = 28.9 ± 3.1	USA	Overweight and obesity	6 months	**IG:** Same as controls plus internet behavior therapy. Additional twenty-four weekly behavioral lessons via email, self-monitoring diaries, and individualised therapist feedback. **CG:** Internet education. One face-to-face group weight loss session and access to a web site with links to weight loss resources.	IG lost more weight than the CG (*p* = 0.005). Changes in waist circumference were also greater in the IG than in the CG at both 3 months (*p* = 0.001) and 6 months (*p* = 0.005).
Follick et al. 1984 [39]	N = 48 **IG:** n = 24 M/W = Not provided Age Mean = Not provided BMI = Not provided **CG:** n = 24 M/W = Not provided Age Mean = Not provided BMI = Not provided	USA	Overweight	18 weeks	**IG:** Weight loss program (14-session behavior modification program) plus incentive procedure. 5$ (×14) deposit was returned (one for each treatment session). **CG:** Weight loss program alone.	Both groups lost weight over the course of the intervention (*p* < 0.001) and there were no significant differences in weight loss between groups. The inclusion of an incentive procedure may improve the effectiveness of a behavioral weight loss intervention by decreasing attrition (*p* < 0.01).

BMI = Body mass index (kg/m^2^); CG = Control group; IG = Intervention group; M/W = Man/Woman; WC = Waist circumference (); WM = Weight management; WM+ = Weight management plus.

**Table 2:** Evaluation of the adequacy of the studies through the 25 assessment items of the CONSORT guide.

**Clinical Trial**	**1**	**2**	**3**	**4**	**5**	**6**	**7**	**8**	**9**	**10**	**11**	**12**	**13**	**14**	**15**	**16**	**17**	**18**	**19**	**20**	**21**	**22**	**23**	**24**	**25**	**Total**	**%**
Thorndike et al. [21]	1	1	0.5	1	1	0.5	0.5	1	0	0	0.5	1	1	1	1	1	1	NA	0	1	1	1	1	1	1	17	70.8
Röhling et al. [22]	1	1	0	1	1	0.5	0.5	1	1	0	0.5	1	1	1	1	1	1	NA	1	1	1	1	1	1	1	19	79.2
Choy et al. [23]	1	1	1	1	1	1	0	0.5	0	0	0	1	0.5	0	1	1	1	NA	0	1	1	1	1	0	0	14.5	60.4
Faghri et al. [24]	0.5	1	0.5	0.5	1	0.5	0	0	0	0	0	0.5	0	0	0	1	1	NA	0	1	1	1	0	0	1	8.5	35.4
Leedo et al. [25]	1	1	1	1	1	1	0.5	1	1	1	1	1	0.5	1	1	1	1	NA	0	1	1	1	1	1	1	21.5	89.6
Østbye et al. [26]	1	1	1	1	1	0.5	0	0.5	0	0	0	0.5	1	1	1	1	1	NA	0	1	1	1	1	1	1	16	66.7
Stites et al. [27]	0.5	1	0.5	1	1	1	0.5	0.5	0.5	0	0	1	1	0.5	1	1	1	NA	0	1	1	1	1	1	1	16	66.7
Christensen et al. [28]	1	1	0.5	0.5	1	0.5	0	0.5	1	1	0.5	0.5	1	1	0	1	1	NA	0	1	1	1	1	1	1	15	62.5
Speroni et al. [29]	0.5	1	0.5	1	1	1	1	NA	NA	NA	NA	0	0.5	0.5	1	1	1	NA	0	1	1	1	0	0	0	12.5	52.1
Thorndike et al. [30]	1	1	0	1	0	0.5	0.5	0.5	0	0	0	1	1	1	1	1	1	NA	0	1	1	1	1	1	1	15	62.5
Christensen et al. [31]	1	1	0.5	0.5	1	0.5	0.5	0.5	1	1	0.5	0.5	1	1	0	1	1	NA	0	1	1	1	1	1	1	15	62.5
Lemon et al. [32]	1	1	1	1	1	1	0.5	1	1	0	0	1	1	1	1	1	1	NA	0	1	1	1	0	0	1	18	75.0
Lowe et al. [33]	0.5	1	0.5	1	1	1	1	0.5	1	0	0	1	0.5	0.5	0	1	1	NA	0	1	1	1	0	0	1	14.5	60.4
Racette et al. [34]	0.5	1	0.5	1	1	1	0.5	0.5	0.5	0	0	1	1	0.5	0	1	1	NA	1	1	1	1	0	0	1	14	58.3
van Wier et al. [35]	1	1	1	0.5	0.5	0.5	0.5	0.5	0	0	0	0.5	1	0	1	1	1	NA	0	1	1	1	1	0	1	12	50.0
Tate et al. [36]	0.5	1	1	1	1	1	1	0.5	0	0	0	1	1	0.5	1	1	1	NA	0	1	1	1	0	0	1	16	66.7
Follick et al. [37]	0.5	1	0	0	0	0.5	0	0	0	0	0	0	0.5	0	0	0	0	NA	0	0	0	1	0	0	0	3	12.5

NA = not applied.

**Table 3:** Strategies developed by the reviewed clinical trials to enhance the occupational health of health-care workers with overnutrition.

Strategy (Number of Interventions)	Types of Intervention
1 intervention	- A [23] - C [25] - E [37]
2 interventions	- A + B [36] - A + D [30] - A + E [33]
3 interventions	- A+ B + E [21] - C + D + E [24] - C+ D + F [26] - A + E + F [27] - B + C + D [28,31,32] - A + C + D [29] - A + D + E [34] - A + D + F [35]
4 interventions	- A + C + D + F [22]

**Table 4:** Summary of variation in body weight and body mass index in the reviewed trials.

Body Weight Data [kg]							
Trial	Year	N Initial	M-Weight Initial	SD Initial	N Final	M-Weight Final	SD Final
Thorndike et al. [21]	2021	299	79.8	18.8	255	81.3	17.3
Röhling et al. [22]	2020	15	104.0	25	14	96.2	21.2
Choy et al. [23]	2017	20	71.9	11.6	20	70.9	11.9
Faghri et al. [24]	2017	51	NR	NR	NR	NR	NR
Leedo et al. [25]	2017	59	70.4	10.6	59	70.3	10.8
Østbye et al. [26]	2015	275	NR	NR	215	NR	NR
Stites et al. [27]	2015	10	91.5	16.1	10	NR	NR
Christensen et al. [28]	2012	54	84.2	15.9	54	78.4	15.8
Speroni et al. [29]	2012	108	NR	NR	70	NR	NR
Thorndike et al. [30]	2012	174	76.9	16.8	157	74.7	15.8
Christensen et al. [31]	2011	54	84.3	16	54	80.7	12.2
Lemon et al. [32]	2010	386	NR	NR	299	NR	NR
Lowe et al. [33]	2010	47	85.5	16.2	47	86.3	16.9
Racette et al. [34]	2009	68	92.4	24.9	68	91.6	25.5
van Wier et al. [35]	2009	462	93.4	14.1	459	90.7	13.7
van Wier et al. [35]	2009	464	92.8	14.3	458	91.0	14.2
Tate et al. [36]	2001	46	77.4	9.4	36	73.3	4.5
Follick et al. [37]	1984	24	NR	NR	NR	NR	NR
**Data on body mass index [kg/m^2^]**							
**Trial**	**Year**	**N** **initial**	**M-IMC** **initial**	**SD** **initial**	**N** **final**	**M-IMC** **Final**	**SD** **final**
Thorndike et al. [21]	2021	299	28.6	6.6	255	29.1	2
Röhling et al. [22]	2020	15	35.1	6.9	14	32.6	1.1
Choy et al. [23]	2017	20	28.6	2.8	20	28.2	3.0
Faghri et al. [24]	2017	51	NR	NR	NR	NR	NR
Leedo et al. [25]	2017	59	24.1	3.5	59	NR	NR
Østbye et al. [26]	2015	215	36.9	5.86	215	36.6	2.2
Stites et al. [27]	2015	10	33.9	5	10	NR	NR
Christensen et al. [28]	2012	54	30.7	5.4	54	28.5	5.5
Speroni et al. [29]	2012	70	29.6	6.6	70	29.1	6.5
Thorndike et al. [30]	2012	157	28	5.8	157	27.2	5.4
Christensen et al. [31]	2011	54	30.5	5.4	54	29.2	4.0
Lemon et al. [32]	2010	386	28.4	NR	299	28.9	NR
Lowe et al. [33]	2010	47	NR	NR	NR	NR	NR
Racette et al. [34]	2009	68	34.5	9.7	68	34.1	9.8
van Wier et al. [35]	2009	462	29.5	3.5	NR	NR	NR
van Wier et al. [35]	2009	462	29.5	3.5	NR	NR	NR
Tate et al. [36]	2001	46	29.1	3	36	NR	NR
Follick et al. [37]	1984	24	NR	NR	NR	NR	NR

N = Intervention population; M-Weight = mean body weight in kg; M-IMC = mean body mass index; SD = Standard Deviation; NR = Not reported.

## Data Availability

Not applicable.
